# Does drug dispensing improve the health outcomes of patients attending community pharmacies? A systematic review

**DOI:** 10.1186/s12913-021-06770-0

**Published:** 2021-08-02

**Authors:** Bárbara Pizetta, Lívia Gonçalves Raggi, Kérilin Stancine Santos Rocha, Sabrina Cerqueira-Santos, Divaldo Pereira de Lyra-Jr, Genival Araujo dos Santos Júnior

**Affiliations:** 1 Research Group on Implementation and Integration of Clinical Pharmacy Services in Brazilian Health System (SUS), Department of Pharmacy and Nutrition, Federal University of Espírito Santo, ES Alegre, Brazil; 2 Health Sciences Graduate Program, Graduate Program in Pharmaceutical Sciences, Laboratory of Teaching and Research in Social Pharmacy (LEPFS), Federal University of Sergipe, SE São Cristóvão, Brazil

**Keywords:** Dispensing, Counseling, Pharmacists, Evidence-Based Practice, Outcomes Assessment, Health Outcomes

## Abstract

**Background:**

Drug dispensing is a clinical pharmacy service that promotes access to medicines and their rational use. However, there is a lack of evidence for the impact of drug dispensing on patients’ health outcomes. Thus, the purpose of this study was to assess the influence of drug dispensing on the clinical, humanistic, and economic outcomes of patients attending community pharmacies.

**Methods:**

A systematic literature search was performed in April 2021 using PubMed, Web of Science, Cochrane Library, LILACS, and Open Thesis. Two reviewers screened titles, abstracts, and full-text articles according to the eligibility criteria. Methodological quality was assessed using tools from the Joanna Briggs Institute, and the literature was synthesized narratively.

**Results:**

We retrieved 3,685 articles and included nine studies that presented 13 different outcomes. Regarding the design, they were cross-sectional (*n* = 4), randomized clinical trials (*n* = 4), and quasi-experimental (*n* = 1). A positive influence of drug dispensing on health outcomes was demonstrated through six clinical, four humanistic and three economic outcomes. Eight studies (88,9 %) used intermediate outcomes. The assessment of methodological quality was characterized by a lack of clarity and/or lack of information in primary studies.

**Conclusions:**

Most articles included in this review reported a positive influence of drug dispensing performed by community pharmacists on patients’ health outcomes. The findings of this study may be of interest to patients, pharmacists, decision makers, and healthcare systems, since they may contribute to evidence-based decision-making, strengthening the contribution of community pharmacists to health care.

**Trial registration:**

Registration: PROSPERO CRD42020191701.

**Supplementary Information:**

The online version contains supplementary material available at 10.1186/s12913-021-06770-0.

## Background

Worldwide, the irrational use of medicines is considered a major public health problem [1, 2]. The World Health Organization (WHO) estimates that more than half of all medicines are prescribed, dispensed, or sold inappropriately and that around 50 % of patients fail to take them correctly [3]. This may have negative implications for patients and health systems, such as increased drug-related problems (DRP), hospital admissions due to adverse reactions, drug poisoning, antimicrobial resistance, and financial losses [4–9].

In this context, community pharmacists have a key role within the healthcare system for promoting rational use of medicines [10]. Tsuyuki and colleagues [11] noted that pharmacists see patients somewhere between 1.5 and 10 times more frequently than their primary care physicians. Thus, by providing clinical pharmacy services, community pharmacists can promote the rational use of medicines, closely monitor medication adherence, thereby improving pharmacotherapy and health outcomes of patients [12–15].

Among clinical pharmacy services, drug dispensing has high visibility in community pharmacies; it is accessible and serves a large number of patients seeking prescription drugs [16, 17]. Drug dispensing can be defined as a clinical pharmacy service that ensures the provision of medicines and other health products through the analysis of technical and legal aspects of prescription, assessment of individual health needs, and performance of interventions in the process of medicine use that includes pharmaceutical counseling and documentation of the interventions [18–20].

In recent years, studies have reported the impact of different clinical pharmacy services on the health outcomes of patients, such as medication review [21], medication reconciliation [22], medication synchronization [23], and medication therapy management [24, 25]. However, there is a lack of evidence for the impact of drug dispensing on health outcomes, and studies have mainly addressed certain aspects of this practice, such as the evaluation of the structure, process, and quality of the service [26–30]. Thus, high-quality studies are needed to evaluate the impact of drug dispensing on clinical, humanistic, and economic outcomes of patients. Therefore, this systematic review aimed to assess the influence of drug dispensing on the health outcomes of patients who attended community pharmacies.

## Methods

This systematic review was conducted in accordance with guidelines in the Cochrane Handbook for Systematic Reviews of Interventions [31], Preferred Reporting Items for Systematic Reviews and Meta-Analyses (PRISMA) [32] and Assessing the Methodological Quality of Systematic Reviews (AMSTAR 2) [33]. The systematic review was registered on the PROSPERO database (CRD 42020191701).

### Search strategy

PICO elements were used to address our clinical question, eligibility criteria, and search strategy. PICO represents an acronym for: (P) patient or problem, (I) intervention or exposure, (C) comparison intervention or exposure and (O) outcome of interest. In present study, PICO was as follows: (P) person, patients, or caregivers who received drug dispensing in community pharmacies; (I) drug dispensing performed by pharmacists; (C) not applicable; and (O) health outcomes influenced by dispensing.

A systematic search of the literature was performed in April 2021 using the following databases: PubMed, Web of Science, the Cochrane Library, LILACS, and Open Thesis. The search strategy used standard (MeSH terms) and non-standard terms related to “dispensing,” “pharmaceutical preparations,” “outcome assessment,” “health care,” “pharmacists,” “community pharmacy services,” and “pharmacies.” Each term was grouped through Boolean operators (AND and OR) to their synonyms and subcategories and adapted to each database. Additionally, we manually searched the reference lists of all eligible studies. The databases were searched for publications until April 2021. The complete search strategy is provided in Additional file 1.

### Eligibility criteria

Studies were eligible for inclusion if they met the following criteria: (i) performed in community pharmacies; (ii) evaluated drug dispensing by pharmacists; (iii) evaluated the influence of drug dispensing on clinical, economic, and/or humanistic outcomes for patients’ health; and (iv) were published in English, Portuguese, or Spanish. The following literature and studies were excluded: (i) conference abstracts; (ii) letters to the editor, (iii) literature reviews; (iv) systematic reviews or meta-analyses; v) studies not available in full, vi) results do not separate the intervention of the pharmacist from the intervention of other professionals, and vii) results do not separate dispensing from other services /interventions. Studies were not excluded on the basis of design, year of publication, or methodological quality.

### Study selection

All duplicate studies were excluded. Next, two researchers (B.P. and L.G.R.) independently reviewed the titles and abstracts, and subsequently, full texts were deemed relevant according to the eligibility criteria. The first two steps were performed using the Rayyan tool (http://rayyan.qcri.org) [34]. Any divergence in terms of study selection was judged by a third investigator (G.A.S.J.).

### Analysis of the degree of agreement

Cohen’s kappa index (κ) was used to assess the level of agreement between the two reviewers in the article selection process, adopting a 95 % confidence interval. The agreement between reviewers was based on the following parameters: κ ≤ 0.10 without agreement; κ: 0.11–0.40 weak agreement; κ: 0.41–0.75 good agreement; and κ > 0.75 excellent agreement [35].

### Data collection process

Two reviewers (B.P. and L.G.R.) independently extracted the data from the included articles using a pre-formatted Microsoft® Excel® spreadsheet. The following data were extracted: authors, year of publication, country, study design, number of participants, health outcomes, main results, and limitations or bias described. In the absence of data and/or clarity of the extracted variables, the authors of the included studies were contacted by email. Any divergence in the data extraction was resolved by reaching a consensus reached-through discussion. Articles that did not mention the study design in the methods were classified independently by two authors (G.A.S.J. and K.S.S.R.), and any disagreement was resolved by consensus.

### Definitions adopted in this systematic review

In this study, we considered that the process of dispensing involves technical and clinical components. The first one refers to the analysis of technical and legal aspects of prescription, correct selection of the prescribed medicine or health product to be dispensed, assembling and labeling, checking the accuracy of it and handing out to the patient. The second one involves the patient care process: (i) assessment of individual health needs of the patient, (ii) elaboration of the care plan with the performance of interventions in the process of medicine use that includes pharmaceutical counseling, and (iii) evaluation of the health outcomes of patients. This process should be documented and also involves frequent communication and collaboration with the patient and other health professionals [27, 36–38].

In this study, we adopted concepts that are widely used in studies on quality assessment in health care. We considered “health outcomes” as all measures attempting to describe the effects of care on the health status of patients and the population [39]. Thus, in view of the range of possible “health outcomes,” we refined the classification of “health outcomes” according to the Economic, Clinical, Humanistic Outcomes (ECHO) Model [40]. The ECHO model provides a theoretical framework for systematic planning of outcomes research according to the following classifications: economic outcomes (reduction in health care costs or utilization, such as hospitalizations, emergency department visits, clinic visits, and/or avoided drug costs), clinical outcomes (improved disease or symptom control, and use of health care treatment), and humanistic outcomes (measures of patient satisfaction and patients’ quality of life) [41–44].

We also classified “health outcomes” according to the following taxonomy: final endpoint (direct measure of an effect with a pharmaceutical product or service, e.g., mortality, morbidity, quality of life, patient satisfaction) and intermediate or surrogate endpoints (indirect measure of an effect in situations where a direct measure of effect is not feasible within a reasonable timeframe, e.g., HbA1c for patients with diabetes, and blood pressure for patients with hypertension) [39]. In literature, the terms “endpoint” and “outcome” are often used interchangeably [45]. Finally, in the present review, all definitions of terms and concepts related to “health outcomes” may be of interest to patients, pharmacists, decision makers, and healthcare systems.

### Quality assessment

Three reviewers (B.P., L.G.R., and K.S.S.R.) independently assessed the quality of the included studies. All discrepancies were resolved by consensus. The tools made available by the Joanna Briggs Institute (JBI) were used: JBI’s critical appraisal tools for Analytical Cross-Sectional Studies (eight items) [46], JBI’s critical appraisal tools for Quasi-Experimental Studies (non-randomized experimental studies) (nine items) [47] and JBI’s critical appraisal tools for Randomized Controlled Trials (13 items) [48]. Each item was marked “yes,” if the article met the criteria of the item; “no,” if it did not meet the criteria; “unclear” if sufficient information to make a judgment was lacking; and “Not Applicable,” if the item did not apply to the article.

## Results

### Search and study selection

**Fig. 1:**
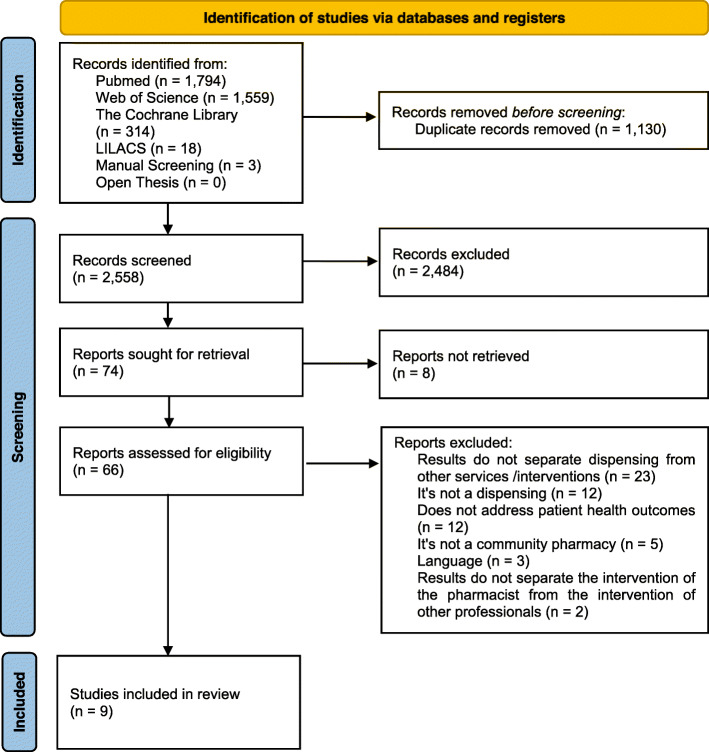
Flow diagram of literature search and screening process. *From*: Page MJ, McKenzie JE, Bossuyt PM, Boutron I, Hoffmann TC, Mulrow CD, et al. The PRISMA 2020 statement: an updated guideline for reporting systematic reviews. BMJ 2021;372:n71. doi: 10.1136/bmj.n71. For more information, visit: http://www.prisma-statement.org/

A total of 3,688 articles were identified from the initial search. After excluding duplicated or irrelevant articles based on titles and abstracts, 74 potentially relevant articles were retrieved for full-text evaluation. Out of these, nine met the inclusion criteria and were included in this systematic review. Figure 1 illustrates the study selection process. There was strong agreement between the two evaluators in their analysis of titles and abstracts (κ1 = 0.892) and in their analysis of full studies (κ2 = 0.962). Articles that were excluded after full-text review and the reasons for exclusion are summarized in Additional file 2.

### Study characteristics

The characteristics of the included studies are shown in Table 1. All nine articles were published in English between 2006 and 2020. The studies were performed on four continents (America, Europe, Oceania, and Asia) and in seven different countries (Ireland, Brazil, Poland, the United Arab Emirates, Sweden, Australia, and the United States of America (USA). Most studies that were included were conducted in Australia (*n* = 3).

**Table 1:** Characteristics of studies included in this systematic review

Study	Country	Design	Sample size	Evaluated outcomes	Main results	Limitations and bias described
Chong et al., 2011 [49]	Australia	Cross-sectional	97 community pharmacists	Cost-savings for patients who accepted a generic substitution.	The average cost-saving per item was AUD$2.26.	Low response rate; survey was time-consuming; pharmacist selection bias; limited representation of the collected brand name prescriptions; the findings cannot be generalized to whole population of community pharmacies.
Basheti et al. 2008 [50]	Australia	Single-blind cluster randomized parallel group design	112 patients	Peak expiratory flow (PEF) variability, asthma severity; asthma-related quality of life.	In the active group, asthma severity was significantly reduced at 2 months (*p* = 0.001), 3 months (*p* < 0.001), and 6 months (*p* = 0.015) compared with the control group. By 3 months, only 8 % of active group patients were classified as having severe asthma compared with 22 % of control group patients (*p* = 0.037). Post-hoc analysis demonstrated a significant correlation between improvement in Inhaler Technique Score and improvement in both PEF variability (*r* = 0.31, *p* = 0.008) and asthma related quality of life( *r* = 0.37, *p* = 0.001).	Not reported.
Crockett et al. 2006 [51]	Australia	Parallel group design with a control and intervention group	106 patients (60 in the control group and 46 in the intervention group)	Patient’s psychological wellbeing (K10 scores), patient satisfaction with service.	Wellbeing was improved in control and intervention groups (the K10 scores decreased significantly from baseline to 2 months in the control and intervention groups by 4 points [*p* < 0.0001] and 4.7 points [*p* ≤ 0.001], respectively). However, the K10 score for the intervention group continued to decrease in the second month whereas that of the control group increased slightly (16.7/50 versus 17.7/50 control [*p* = 0.176]; 20.5/50 versus 18.3/50 [*p* = 0.009] intervention).	The project was of insufficient duration to effectively measure the full effect of the pharmacists’ intervention; telephone contact and K10 administration might have had beneficial effects and masked the impact of pharmacists in the intervention group; problems were experienced with the video-conference link, which might have limited the efficacy of training.
Ali et al. 2019 [52]	United Arab Emirates	Cross-sectional	210 patients	Patients’ perception towards pharmacist’ performance and satisfaction with dispensing related to privacity.	A total of 136 (64.7 %) patients strongly agreed or agreed that they were satisfied with the pharmacist counselling regarding the questions asked before dispensing medications, such as history of previous drug allergy, disease details, etc. 193 (91.9 %) patients disagreed or strongly disagreed that they were satisfied with the privacy maintained by pharmacist while discussing with patients and dispensing medications.	The results of this study cannot be generalized to all patients in Dubai; limited numbers of participants; closed-ended questions in the questionnaire may not help to clarify expectations.
Merks et al. 2019 [53]	Poland	Randomized trial	199 patients (102 in control group and 97 in intervention group)	Relief of symptoms after antibiotic therapy.	A total of 89 patients (91.7 %) in the intervention group (pharmacy practice with pictograms) and 86 patients (84.3 %) in the control group (usual pharmacy practice) reported symptom relief after antibiotic therapy (OR: 2.07, 95 % CI: 0.84–5.08, *p* = 0.1127). Regarding the subjective assessment of patients’ perspective, the net promotor score was higher for the intervention group (71.3 %, *n* = 69) than for the control group (51.5 %, *n* = 52). The chance that a patient was an advocate of pharmaceutical services was twice as likely in the case of pharmaceutical practice supported by pictograms (OR 2.04; 95 % CI 1.09 3.81; *p* = 0.0239).	Limited number of patients; authorial questionnaires were only validated in terms of face and content validity; subjectivity in outcome selection; psychological bias in patients’ responses (due to the interviewers being pharmacists); participation bias due to the randomized cluster design (patient randomization was not possible); duration of the study was relatively short.
O’Dwyer et al. 2020 [54]	Ireland	Cluster randomized open-label clinical trial	152 patients (74 in the biofeedback group, 56 in the demonstration group, and 22 in the control group)	Quality of life; self-reported respiratory symptom (presence of cough, breathlessness, and nighttime symptoms); asthma exacerbation rate.	In both the biofeedback and demonstration groups, there were statistically significant reductions in the total quality of life scores from month 1 to month 2, with decreases of 5.3 and 5.7, respectively. However, only patients in the biofeedback group had a sustained reduction to month 6 (-6.1; 95 % CI, -9.68 to -0.4; *p* = 0.04). There was a decrease in daily respiratory symptoms in the biofeedback group. Over the 6-month study period there was a numerically smaller number of moderate exacerbations of airway disease treated with both antibiotics and oral corticosteroids in the biofeedback group, 0.7 (95 % CI, 0.4–1.1) compared with 1.1 (95 % CI, 0.5–1.7) in the demonstration group, and 0.9 (95 % CI, 0.1–1.7) in the control group.	The cluster randomized design can allow significant imbalances between groups to occur at baseline. There was an interval of 3 months where all study procedures ceased. Recruitment was only 80 % of original target. Small number of patients per cluster and high dropout rate in the second half of the study.
Westerlund, 2009 [55]	Sweden	Cross-sectional^a^	89 community pharmacies	Economic outcomes of community pharmacy interventions in patients’ drug-related problems (DRP).	Pharmacy interventions have saved 68 (13 %) primary care contacts and 16 (3 %) future hospitalizations. The potential societal cost savings extrapolated to Sweden on the national level were estimated to be €358 million.	DRP were assessed and interventions were documented by one pharmacist and one physician; a relatively small number of DRP and interventions were evaluated; extrapolation to the national level in Sweden should be interpreted as demonstrating the potential societal cost savings by pharmacy interventions rather than as showing actual cost savings.
Payne et al., 2019 [56]	United States of America	Cross-sectional^a^	200 patients	Total money saved with dispensing services for each averted adverse event.	The estimated cost savings for each adverse event avoided resulted in a minimum cost savings of $10,458.	The study was performed in one independent pharmacy. The intervention patterns in this pharmacy are not generalizable; a retrospective data analysis was performed, making it unclear whether the annotations resulted in further activities that increased patient safety; the exact clinical outcome of each individual activity is unknown; data were collected over a 2-month period.
Ferreira et al., 2018 [57]	Brazil	Pre-post-test design (quasi-experimental^a^)	104 patients	Patient satisfaction with service.	Patients reported a high level of satisfaction towards the dispensing service, which was rated excellent or very good by more than 70 % of the patients.	The Morisky scale used in the study tends to overestimate non-adherence behavior; satisfaction may be overestimated, particularly considering that the patients interviewed were frequent users of the University Pharmacy.

*AUD* Australian dollar, *CI* confidence interval, *DRP* drug-related problems, *Or* odds ration, *PEF* peak expiratory flow

^a^Classification of study design by the authors of this systematic review

The articles reported the following design: cross-sectional (*n* = 4) [49, 52, 55, 56], randomized clinical trials (*n* = 4) [50, 51, 53, 54] and quasi-experimental (*n* = 1) [57]. The study sample was heterogeneous and varied in terms of number of patients (104–210) and community pharmacies (89–97). There was also heterogeneity in patient characteristics between studies, with the majority of them not specifying the patients’ characteristics or conditions (*n* = 5). The remaining four studies stated that patients used antibiotics (*n* = 1), antidepressants (*n* = 1) or inhaled drugs (*n* = 2).

### Influence of drug dispensing on health outcomes

Thirteen different health outcomes were assessed in primary studies. Out of the four studies that evaluated clinical outcomes, two of them were related to the impact of dispensing on the care of patients with asthma [50, 54]. The more reported humanistic outcomes were satisfaction (*n* = 3) [51, 52, 57] and quality of life (*n* = 2) [50, 54]. Three economic outcomes were assessed in three studies, and the most frequent outcome was cost-saving (*n* = 3) [49, 55, 56]. Most studies evaluated intermediate endpoints [50–56]. In addition, health outcomes were measured heterogeneously in the included studies. Health outcomes, their classifications, and how they were measured are presented in Table 2.

**Table 2:** Measures used to assess clinical, economic, and humanistic outcomes

ECHO Model	Type		Health outcomes	How was it measured?	Reference
	**I**	**F**			
**Clinical**	**X**		Peak expiratory flow (PEF).	Peak flow variability was calculated as Min%Max (lowest morning PEF over two weeks, as a percentage of highest PEF over the same period).	Basheti et al., 2008 [50]
	**X**		Asthma severity.	Asthma severity was categorized based on the Australian Asthma Management Handbook.	Basheti et al., 2008 [50]
	**X**		Patients’ psychological wellbeing.	Interviewed patients answered the Kessler Psychological Distress Scale (K10).	Crockett et al., 2006 [51]
	**X**		Relief of symptoms after antibiotic therapy.	Semi-structured interview based on questionnaires prepared by a member of the research team and tested for face and content validity during the pilot study.	Merks et al., 2019 [53]
	**X**		Respiratory symptoms.	Symptoms, such as cough, breathlessness, and night-time symptoms, were recorded daily in a diary by the biofeedback and demonstration groups.	O’Dwyer et al., 2020 [54]
	**X**		Asthma exacerbation.	Exacerbations were assessed by quantifying episodes when either oral corticosteroids and/or antibiotics usually indicated for respiratory infection were dispensed.	O’Dwyer et al., 2020 [54]
**Economic**		**X**	Cost savings.	The cost-savings achieved for patients by accepting generic substitutions were determined based on the dispensed prices to the patient for branded medicines and relevant generic substitutes listed on the Schedule of Pharmaceutical Benefits Scheme (PBS).	Chong et al., 2011 [49]
	**X**		Cost savings.	The direct costs to society in terms of health care resources needed to respond to the DRP (i.e., cost for primary care visits and hospitalizations) that were potentially avoided as a result of the interventions, were calculated and extrapolated to the national level on an annual basis.	Westerlund et al., 2009 [55]
	**X**		Primary care contact avoided.	The authors of the study, a pharmacist and a physician with extensive clinical experience, judged in terms of primary care contacts avoided.	Westerlund et al., 2009 [55]
	**X**		Hospitalization avoided.	The authors of the study, a pharmacist and a physician with extensive clinical experience, judged in terms of hospitalization avoided.	Westerlund et al., 2009 [55]
	**X**		Money saved.	A literature review was conducted to determine associated cost.	Payne et al., 2019 [56]
**Humanistic**		**X**	Asthma-related quality of life.	Not reported	Basheti et al., 2008 [50]
		**X**	Patient satisfaction with service.	Not reported	Crockett et al., 2006 [51]
	**X**		Patients’ perception towards pharmacist’ performance.	Questionnaire based on themes of previous studies. The questionnaire was validated and based on a 5-point Likert type scale with responses ranging from strongly agrees, to strongly disagree.	Ali et al., 2019 [52]
	**X**		Satisfaction with the privacy maintained by pharmacist.	Questionnaire based on themes of previous studies. The questionnaire was validated and based on a 5-point Likert type scale with responses ranging from strongly agrees, to strongly disagree.	Ali et al., 2019 [52]
	**X**		Subjective assessment of patients’ perspective on medical information relating to antibiotic therapy.	Patients’ perspective was measured using the Net Promoter Score Calculation, provided in the form of a single question that aimed to assess how willing a consumer is to recommend a particular product to other users.	Merks et al., 2019 [53]
		**X**	Quality of life.	Quality of life was measured by the St George’s Respiratory Questionnaire.	O’Dwyer et al., 2020 [54]
		**X**	Patient satisfaction with service.	Patients’ satisfaction with the drug dispensing service was assessed by an appropriate instrument validated in English and translated into Portuguese (Correr Instrument).	Ferreira et al., 2018 [57]

*F *Final endpoint, *I* intermediate endpoint, *K10* Kessler Psychological Distress Scale, *Min%Max* lowest (Min) and the highest (Max) value, *DRP* Drug-related problem

### Quality assessment

The methodological quality of the studies is presented in Additional file 3. Regarding cross-sectional studies, two of them (50 %) [49, 52] met at least five of the eight evaluated criteria. The quasi-experimental study met five of the nine evaluated criteria[57]. Only one randomized clinical trial met six of the thirteen criteria evaluated [54], and the remaining clinical trials met four or fewer criteria [50, 51, 53].

## Discussion

The results of this systematic review demonstrate the positive influence of drug dispensing on the health outcomes of patients attending community pharmacies. Studies that address drug dispensing usually assess the quality of the practice showing a non-systematized and undocumented process [26, 30, 58]. Several strategies have been proposed to qualify this service, such as the development of instruments to support the practice and training pharmacists and pharmacy teams [27, 38, 59]. Another important point to be considered in quality assessment is knowing which health outcomes can be impacted by the services [39]. Thus, our results can help pharmacists to measure the influence of their practice on patients’ health. In addition, the results of this review can be used by policymakers and stakeholders to support public policies and other interventions that improve drug dispensing.

Regarding the influence of drug dispensing on clinical outcomes, most articles included intermediate endpoints. These findings may be related to the characteristics of drug dispensing as a fast service that supports many people, making it difficult to adopt designs that use the final endpoints [17, 60]. In addition, these results may reflect the purpose of drug dispensing in promoting access to medicines and their rational use. Thus, interventions by pharmacists are usually focused on improving the process of medication use by the patient [61, 62]. Besides, half of the studies that evaluated clinical outcomes were asthma related. The role of the pharmacist in the management of asthma is already well documented in the literature [63, 64]. Studies have shown that the most common pharmacists’ interventions on health outcomes in patients with asthma were patient counseling [65, 66]. Thus, since patient counseling is a core component of the drug dispensing, the service may contribute to the care of patients with asthma.

In relation to humanistic outcomes, studies have reported improved quality of life and patient satisfaction with drug dispensing service. Similarly, a randomized controlled trial performed in community pharmacies and cooperative general practices in the Netherlands showed that health-related quality of life in older patients increased (95 % CI: 0.94 to 5.8; *p* = 0.006) with use of a clinical medication review service [67]. In addition, systematic reviews have also revealed a high level of patient satisfaction with clinical pharmacy services in community pharmacies thus increasing customer loyalty [68, 69]. Analyzing these health outcomes is important for improving the quality of clinical pharmacy services [70]. Thereby, given the importance of humanistic outcomes for patient health, further studies are needed to assess the influence of drug dispensing on this outcome to expand and reinforce this evidence.

With respect to economic outcomes, few studies have evaluated the influence on reducing costs for patients and/or health systems. A systematic review that assessed the impact of interventions by community pharmacists on the management of chronic obstructive pulmonary disease reported similar results [71]. Although economic evaluation is challenging, in recent years, research in this field has expanded in the health sciences [72, 73]. The International Society for Pharmacoeconomics and Outcomes Research has highlighted the importance of evaluating the economic outcomes of health services to reduce costs and improve decision-making regarding investments [74, 75]. Therefore, further studies are needed to assess the economic impact of clinical pharmacy services, including drug dispensing.

Regarding the assessment of methodological quality, a lack of clarity and/or an absence of information were identified in the studies included in this systematic review. These factors, coupled with a lack of consensus on the most appropriate tool to be used as well as the subjectivity of the evaluators in the use of these tools, can impact assessment of methodological quality [76, 77]. In addition, the research must be reported clearly so that readers can understand what was planned, how the research was carried out, and the results and conclusions, to enable data interpretation and reproducibility [78, 79]. Therefore, quality assessment tools to assist in methodological planning and reporting guidelines for the execution and reporting of the study are necessary.

This systematic review has several strengths and limitations. Meta-analysis could not be performed due to difficulties in combining the primary studies as they showed differences in populations and outcome measures. This makes it difficult to interpret our results and can impair the quality of the evidence generated. Furthermore, the quality assessment may have been influenced by the subjectivity of the researchers in the use of the quality assessment tools. We minimized this limitation by including three independent researchers. Thus, these limitations can be overcome by planning and carrying out future primary studies that are methodologically adequate and clearly reported. Conversely, to our knowledge, this is the first systematic review to identify health outcomes that are influenced by drug dispensing in community pharmacies. The findings of this review have the potential to be applied in clinical practice and can be used by researchers to guide studies of high scientific evidence evaluating the impact of drug dispensing and improving health outcomes, thus contributing to evidence-based decision-making. This strengthens efforts to promote rational use of medicines and to minimize the associated risks. In addition, we followed a rigorous methodological process: five different databases were searched using standardized and non-standardized terms; the references of the included studies were searched manually; and articles selection and data extraction were performed by two independent researchers, and a third was consulted in the event of a disagreement. Finally, articles were not excluded based on the methodological quality or study design.

## Conclusions

This systematic review provides insight into the health outcomes that may be influenced by the drug dispensing provided by community pharmacists and revealed that most studies reported positive results. Most of the studied health outcomes were intermediate endpoints, with emphasis on clinical outcomes asthma related, cost savings, and satisfaction. A limited number of studies have assessed the influence of drug dispensing on health outcomes, addressing the need for further research in this field. Thus, policymakers and stakeholders may encourage public policies and develop interventions that encourage the qualification of drug dispensing in community pharmacies. We also observed heterogeneity in the tools used to measure health outcomes. We suggest that future studies, with high scientific evidence and methodological quality, should assess the outcomes influenced by drug dispensing identified in this review to strengthen the evidence for these services. Our results may also be useful for developing strategies to improve drug dispensing practice and, consequently, patients’ health outcomes to ensure rational medicine use and patient safety.

## Supplementary Information

**Additional file 1. ** Database search strategy.

**Additional file 2. ** List of excluded articles and reason for exclusion.

**Additional file 3. ** Assessment of methodological quality

## Data Availability

All data generated or analysed during this study are included in this published article and its supplementary information files.

## Abbreviations

WHO: World Health Organization
DRP: Drug-Related Problems
PRISMA: Preferred Reporting Items for Systematic Reviews and Meta-Analyses
AMSTAR: Assessing the Methodological Quality of Systematic Review
ECHO: Economic, Clinical, Humanistic Outcomes
JBI: Joanna Briggs Institute
USA: United States of America
CAPES: Coordenação de Aperfeiçoamento de Pessoal de Nível Superior – Brasil
FAPES: Foundation for Research Support of Espírito Santo
SUS: Brazilian Health System
