# Supplementary material for: Does drug dispensing improve the health outcomes of patients attending community pharmacies? A systematic review
Source: BMC Health Serv Res. 2021 Aug 2;21:764. doi: 10.1186/s12913-021-06770-0 (PMC8330087; doi:10.1186/s12913-021-06770-0)
Supplement: Supplementary file 1 — Additional file 1. Database search strategy. [file 12913_2021_6770_MOESM1_ESM.docx]

**Does drug dispensing improve the health outcomes of patients attending community pharmacies? A systematic review**

**Bárbara Pizetta**

Research Group on Implementation and Integration of Clinical Pharmacy Services in Brazilian Health System (SUS), Department of Pharmacy and Nutrition, Federal University of Espírito Santo, Alegre, ES, Brazil.

E-mail: pizetta.barbara@gmail.com

**Lívia Gonçalves Raggi**

Research Group on Implementation and Integration of Clinical Pharmacy Services in Brazilian Health System (SUS), Department of Pharmacy and Nutrition, Federal University of Espírito Santo, Alegre, ES, Brazil.

E-mail: livia_gr@hotmail.com

**Kérilin Stancine Santos Rocha**

Health Sciences Graduate Program

Laboratory of Teaching and Research in Social Pharmacy (LEPFS), Department of Pharmacy, Federal University of Sergipe, São Cristóvão, SE, Brazil.

E-mail: kerilin.farm@gmail.com

**Sabrina Cerqueira Santos**

Graduate Program in Pharmaceutical Sciences

Laboratory of Teaching and Research in Social Pharmacy (LEPFS), Department of Pharmacy, Federal University of Sergipe, São Cristóvão, SE, Brazil.

E-mail: Sabrina-cerqueira@hotmail.com

**Divaldo Pereira de Lyra Jr**

Laboratory of Teaching and Research in Social Pharmacy (LEPFS), Department of Pharmacy, Federal University of Sergipe, São Cristóvão, SE, Brazil.

E-mail: lepfs.ufs@gmail.com

**Genival Araujo dos Santos Júnior***

Research Group on Implementation and Integration of Clinical Pharmacy Services in Brazilian Health System (SUS), Department of Pharmacy and Nutrition, Federal University of Espírito Santo, Alegre, ES, Brazil.

E-mail: farm.genival@gmail.com

***Corresponding author:**

**Genival Araujo dos Santos Júnior***

Research Group on Implementation and Integration of Clinical Pharmacy Services in Brazilian Health System (SUS), Department of Pharmacy and Nutrition, Federal University of Espírito Santo, Alegre, ES, Brazil.

E-mail: [farm.genival@gmail.com](about:blank)

**Appendix A - Database search strategy**

| *Pubmed* | #1 | Search: ((((((((((((("Dispensing") OR ("Drug dispensing")) OR ("Medication dispensing")) OR ("Medicine dispensing")) OR ("Drugs dispensing")) OR ("Medications dispensing")) OR ("Medicines dispensing")) OR ("counselling")) OR ("counseling")) OR ("Advice")) |
| --- | --- | --- |
|  | #2 | Search: (("Pharmaceutical Preparations"[MeSH Terms]) OR (((((((((((((((((("Preparations, Pharmaceutical") OR ("Pharmaceutic Preparations")) OR ("Preparations, Pharmaceutic")) OR ("Pharmaceutical Products")) OR ("Products, Pharmaceutical")) OR ("Pharmaceuticals")) OR ("Drugs")) OR ("Drug")) OR ("Medicine")) OR ("Medicines")) OR ("Medication")) OR ("Medications")) OR ("OTC Drug")) OR ("Drug, OTC")) OR ("Over the Counter Drug")) OR ("Patent Medicine")) OR ("Over-the-Counter Drug")) OR ("Pharmaceutical Preparations")))) |
|  | #3 | Search: (("Outcome Assessment, Health Care"[MeSH Terms]) OR (((((((((((((((((((((((((("Outcomes Assessment") OR ("Outcome Assessment (Health Care)")) OR ("Assessment, Outcome (Health Care)")) OR ("Assessments, Outcome (Health Care)")) OR ("Outcome Assessments (Health Care)")) OR ("Assessment, Outcomes")) OR ("Assessments, Outcomes")) OR ("Outcomes Assessments")) OR ("Outcomes Research")) OR ("Research, Outcomes")) OR ("Outcome Studies")) OR ("Outcome Study")) OR ("Studies, Outcome")) OR ("Study, Outcome")) OR ("Outcome Measures")) OR ("Measure, Outcome")) OR ("Measures, Outcome")) OR ("Outcome Measure")) OR ("Outcome")) OR ("Outcomes")) OR ("Result")) OR ("Results")) OR ("Health outcome")) OR ("Health outcomes")) OR ("Therapeutic outcome")) OR ("Outcome Assessment, Health Care")))) |
|  | #4 | Search: (("Pharmacists"[MeSH Terms]) OR (((((((((((((("Pharmacist") OR ("Clinical Pharmacists")) OR ("Clinical Pharmacist")) OR ("Pharmacist, Clinical")) OR ("Pharmacists, Clinical")) OR ("Community Pharmacists")) OR ("Community Pharmacist")) OR ("Pharmacist, Community")) OR ("Pharmacists, Community")) OR ("Retail Pharmacists")) OR ("Pharmacist, Retail")) OR ("Pharmacists, Retail")) OR ("Retail Pharmacist")) OR ("Pharmacists")))) OR ((("community pharmacy services"[MeSH Terms]) OR ((((((((((((((((("Pharmaceutical Service, Community") OR ("Pharmaceutical Services, Community")) OR ("Service, Community Pharmaceutical")) OR ("Services, Community Pharmaceutical")) OR ("Pharmacy Services, Community")) OR ("Community Pharmacy Service")) OR ("Pharmacy Service, Community")) OR ("Services, Community Pharmaceutic")) OR ("Services, Community Pharmacy")) OR ("Community Pharmaceutic Services")) OR ("Community Pharmaceutic Service")) OR ("Pharmaceutic Service, Community")) OR ("Pharmaceutic Services, Community")) OR ("Service, Community Pharmaceutic")) OR ("Community Pharmaceutical Services")) OR ("Community Pharmaceutical Service")) OR ("Service, Community Pharmacy"))) OR (("pharmacies"[MeSH Terms]) OR (((((((("Pharmacy Distribution") OR ("Distribution, Pharmacy")) OR ("Distributions, Pharmacy")) OR ("Pharmacy Distributions")) OR ("Community Pharmacies")) OR ("Community Pharmacy")) OR ("Pharmacies, Community")) OR ("Pharmacy, Community")))) |
|  | #5 | ((((((((((((("Dispensing") OR ("Drug dispensing")) OR ("Medication dispensing")) OR ("Medicine dispensing")) OR ("Drugs dispensing")) OR ("Medications dispensing")) OR ("Medicines dispensing")) OR ("counselling")) OR ("counseling")) OR ("Advice")) AND (("Pharmaceutical Preparations"[MeSH Terms]) OR (((((((((((((((((("Preparations, Pharmaceutical") OR ("Pharmaceutic Preparations")) OR ("Preparations, Pharmaceutic")) OR ("Pharmaceutical Products")) OR ("Products, Pharmaceutical")) OR ("Pharmaceuticals")) OR ("Drugs")) OR ("Drug")) OR ("Medicine")) OR ("Medicines")) OR ("Medication")) OR ("Medications")) OR ("OTC Drug")) OR ("Drug, OTC")) OR ("Over the Counter Drug")) OR ("Patent Medicine")) OR ("Over-the-Counter Drug")) OR ("Pharmaceutical Preparations")))) AND (("Outcome Assessment, Health Care"[MeSH Terms]) OR (((((((((((((((((((((((((("Outcomes Assessment") OR ("Outcome Assessment (Health Care)")) OR ("Assessment, Outcome (Health Care)")) OR ("Assessments, Outcome (Health Care)")) OR ("Outcome Assessments (Health Care)")) OR ("Assessment, Outcomes")) OR ("Assessments, Outcomes")) OR ("Outcomes Assessments")) OR ("Outcomes Research")) OR ("Research, Outcomes")) OR ("Outcome Studies")) OR ("Outcome Study")) OR ("Studies, Outcome")) OR ("Study, Outcome")) OR ("Outcome Measures")) OR ("Measure, Outcome")) OR ("Measures, Outcome")) OR ("Outcome Measure")) OR ("Outcome")) OR ("Outcomes")) OR ("Result")) OR ("Results")) OR ("Health outcome")) OR ("Health outcomes")) OR ("Therapeutic outcome")) OR ("Outcome Assessment, Health Care")))) AND (("Pharmacists"[MeSH Terms]) OR (((((((((((((("Pharmacist") OR ("Clinical Pharmacists")) OR ("Clinical Pharmacist")) OR ("Pharmacist, Clinical")) OR ("Pharmacists, Clinical")) OR ("Community Pharmacists")) OR ("Community Pharmacist")) OR ("Pharmacist, Community")) OR ("Pharmacists, Community")) OR ("Retail Pharmacists")) OR ("Pharmacist, Retail")) OR ("Pharmacists, Retail")) OR ("Retail Pharmacist")) OR ("Pharmacists")))) AND ((("community pharmacy services"[MeSH Terms]) OR ((((((((((((((((("Pharmaceutical Service, Community") OR ("Pharmaceutical Services, Community")) OR ("Service, Community Pharmaceutical")) OR ("Services, Community Pharmaceutical")) OR ("Pharmacy Services, Community")) OR ("Community Pharmacy Service")) OR ("Pharmacy Service, Community")) OR ("Services, Community Pharmaceutic")) OR ("Services, Community Pharmacy")) OR ("Community Pharmaceutic Services")) OR ("Community Pharmaceutic Service")) OR ("Pharmaceutic Service, Community")) OR ("Pharmaceutic Services, Community")) OR ("Service, Community Pharmaceutic")) OR ("Community Pharmaceutical Services")) OR ("Community Pharmaceutical Service")) OR ("Service, Community Pharmacy"))) OR (("pharmacies"[MeSH Terms]) OR (((((((("Pharmacy Distribution") OR ("Distribution, Pharmacy")) OR ("Distributions, Pharmacy")) OR ("Pharmacy Distributions")) OR ("Community Pharmacies")) OR ("Community Pharmacy")) OR ("Pharmacies, Community")) OR ("Pharmacy, Community"))) |
| *Cochrane library* | #1 | "Dispensing" OR "Drug dispensing" OR "Medication dispensing" OR "Medicine dispensing" OR "Drugs dispensing" OR "Medications dispensing" OR "Medicines dispensing" OR "counselling" OR "counseling" OR "Advice" |
|  | #2 | MeSH descriptor: [Pharmaceutical Preparations] explode all trees |
|  | #3 | "Preparations, Pharmaceutic" OR "Pharmaceutical Products" OR "Products, Pharmaceutical" OR "Pharmaceuticals" OR "Drugs" OR "Drug" OR "Medicine" OR "Medicines" OR "Medication" OR "Medications" OR "OTC Drug" OR "Drug, OTC" OR "Over the Counter Drug" OR "Patent Medicine" OR "Over-the-Counter Drug" OR "Pharmaceutical Preparations" |
|  | #4 | #2 OR #3 |
|  | #5 | MeSH descriptor: [Pharmacists] explode all trees |
|  | #6 | "Pharmacists" OR "Pharmacist" OR "Clinical Pharmacists" OR "Clinical Pharmacist" OR "Pharmacist, Clinical" OR "Pharmacists, Clinical" OR "Community Pharmacists" OR "Community Pharmacist" OR "Pharmacist, Community" OR "Pharmacists, Community" OR "Retail Pharmacists" OR "Pharmacist, Retail" OR "Pharmacists, Retail" OR "Retail Pharmacist" OR "Pharmacists" OR "Pharmacist" OR "Clinical Pharmacists" OR "Clinical Pharmacist" OR "Pharmacist, Clinical" OR "Pharmacists, Clinical" OR "Community Pharmacists" OR "Community Pharmacist" OR "Pharmacist, Community" OR "Pharmacists, Community" OR "Retail Pharmacists" OR "Pharmacist, Retail" OR "Pharmacists, Retail" OR "Retail Pharmacist" OR "Pharmacists" |
|  | #7 | #5 OR #6 |
|  | #8 | MeSH descriptor: [Outcome Assessment, Health Care] explode all trees |
|  | #9 | "Outcome Assessment, Health Care" OR "Outcomes Assessment" OR "Outcome Assessment Health Care " OR "Assessment, Outcome Health Care " OR "Assessments, Outcome Health Care " OR "Outcome Assessments Health Care " OR "Assessment, Outcomes" OR "Assessments, Outcomes" OR "Outcomes Assessments" OR "Outcomes Research" OR "Research, Outcomes" OR "Outcome Studies" OR "Outcome Study" OR "Studies, Outcome" OR "Study, Outcome" OR "Outcome Measures" OR "Measure, Outcome" OR "Measures, Outcome" OR "Outcome Measure" OR "Outcome" OR "Outcomes" OR "Result" OR "Results" OR "Health outcome" OR "Health outcomes" OR "Therapeutic outcome" OR "Outcome Assessment, Health Care" |
|  | #10 | #8 OR #9 |
|  | #11 | MeSH descriptor: [Community Pharmacy Services] explode all trees |
|  | #12 | MeSH descriptor: [Pharmacies] explode all trees |
|  | #13 | "community pharmacy services" OR "Pharmaceutical Service, Community" OR "Pharmaceutical Services, Community" OR "Service, Community Pharmaceutical" OR "Services, Community Pharmaceutical" OR "Pharmacy Services, Community" OR "Community Pharmacy Service" OR "Pharmacy Service, Community" OR "Services, Community Pharmaceutic" OR "Services, Community Pharmacy" OR "Community Pharmaceutic Services" OR "Community Pharmaceutic Service" OR "Pharmaceutic Service, Community" OR "Pharmaceutic Services, Community" OR "Service, Community Pharmaceutic" OR "Community Pharmaceutical Services" OR "Community Pharmaceutical Service" OR "Service, Community Pharmacy" OR "pharmacies" OR "Pharmacy Distribution" OR "Distribution, Pharmacy" OR "Distributions, Pharmacy" OR "Pharmacy Distributions" OR "Community Pharmacies" OR "Community Pharmacy" OR "Pharmacies, Community" OR "Pharmacy, Community" |
|  | #14 | #11 OR #12 OR #13 |
|  | #15 | #1 AND # 4 AND #7 AND #10 AND #14 |
| *LILACS* | #1 | "Dispensing" OR "Drug dispensing" OR "Medication dispensing" OR "Medicine dispensing" OR "Drugs dispensing" OR "Medications dispensing" OR "Medicines dispensing" OR "counselling" OR "counseling" OR "Advice" AND "Pharmaceutical Preparations" OR "Preparations, Pharmaceutical" OR "Pharmaceutic Preparations" OR "Preparations, Pharmaceutic" OR "Pharmaceutical Products" OR "Products, Pharmaceutical" OR "Pharmaceuticals" OR "Drugs" OR "Drug" OR "Medicine" OR "Medicines" OR "Medication" OR "Medications" OR "OTC Drug" OR "Drug, OTC" OR "Over the Counter Drug" OR "Patent Medicine" OR "Over-the-Counter Drug" OR "Pharmaceutical Preparations" AND "Outcome Assessment, Health Care" OR "Outcomes Assessment" OR "Outcome Assessment Health Care " OR "Assessment, Outcome Health Care " OR "Assessments, Outcome Health Care " OR "Outcome Assessments Health Care " OR "Assessment, Outcomes" OR "Assessments, Outcomes" OR "Outcomes Assessments" OR "Outcomes Research" OR "Research, Outcomes" OR "Outcome Studies" OR "Outcome Study" OR "Studies, Outcome" OR "Study, Outcome" OR "Outcome Measures" OR "Measure, Outcome" OR "Measures, Outcome" OR "Outcome Measure" OR "Outcome" OR "Outcomes" OR "Result" OR "Results" OR "Health outcome" OR "Health outcomes" OR "Therapeutic outcome" OR "Outcome Assessment, Health Care" AND "Pharmacists" OR "Pharmacist" OR "Clinical Pharmacists" OR "Clinical Pharmacist" OR "Pharmacist, Clinical" OR "Pharmacists, Clinical" OR "Community Pharmacists" OR "Community Pharmacist" OR "Pharmacist, Community" OR "Pharmacists, Community" OR "Retail Pharmacists" OR "Pharmacist, Retail" OR "Pharmacists, Retail" OR "Retail Pharmacist" OR "Pharmacists" OR "Pharmacist" OR "Clinical Pharmacists" OR "Clinical Pharmacist" OR "Pharmacist, Clinical" OR "Pharmacists, Clinical" OR "Community Pharmacists" OR "Community Pharmacist" OR "Pharmacist, Community" OR "Pharmacists, Community" OR "Retail Pharmacists" OR "Pharmacist, Retail" OR "Pharmacists, Retail" OR "Retail Pharmacist" OR "Pharmacists" AND "community pharmacy services" OR "Pharmaceutical Service, Community" OR "Pharmaceutical Services, Community" OR "Service, Community Pharmaceutical" OR "Services, Community Pharmaceutical" OR "Pharmacy Services, Community" OR "Community Pharmacy Service" OR "Pharmacy Service, Community" OR "Services, Community Pharmaceutic" OR "Services, Community Pharmacy" OR "Community Pharmaceutic Services" OR "Community Pharmaceutic Service" OR "Pharmaceutic Service, Community" OR "Pharmaceutic Services, Community" OR "Service, Community Pharmaceutic" OR "Community Pharmaceutical Services" OR "Community Pharmaceutical Service" OR "Service, Community Pharmacy" OR "pharmacies" OR "Pharmacy Distribution" OR "Distribution, Pharmacy" OR "Distributions, Pharmacy" OR "Pharmacy Distributions" OR "Community Pharmacies" OR "Community Pharmacy" OR "Pharmacies, Community" OR "Pharmacy, Community" |

| *Web of Science* | #1 | TOPIC: ("Dispensing") OR TOPIC: ("Drug dispensing") OR TOPIC: ("Medication dispensing") OR TOPIC: ("Medicine dispensing") OR TOPIC: ("Drugs dispensing") OR TOPIC: ("Medications dispensing") OR TOPIC: ("Medicines dispensing") OR TOPIC: ("counselling") OR TOPIC: ("counseling") OR TOPIC: ("Advice")  Indexes=SCI-EXPANDED, SSCI, A&HCI, CPCI-S, CPCI-SSH, ESCI Timespan=All years |
| --- | --- | --- |
|  | #2 | TOPIC: ("Pharmaceutical Preparations") OR TOPIC: ("Preparations, Pharmaceutical") OR TOPIC: ("Pharmaceutic Preparations") OR TOPIC: ("Preparations, Pharmaceutic") OR TOPIC: ("Pharmaceutical Products") OR TOPIC: ("Products, Pharmaceutical") OR TOPIC: ("Pharmaceuticals") OR TOPIC: ("Drugs") OR TOPIC: ("Drug") OR TOPIC: ("Medicine") OR TOPIC: ("Medicines") OR TOPIC: ("Medication") OR TOPIC: ("Medications") OR TOPIC: ("OTC Drug") OR TOPIC: ("Drug, OTC") OR TOPIC: ("Over the Counter Drug") OR TOPIC: ("Patent Medicine") OR TOPIC: ("Over-the-Counter Drug") OR TOPIC: ("Pharmaceutical Preparations")  Indexes=SCI-EXPANDED, SSCI, A&HCI, CPCI-S, CPCI-SSH, ESCI Timespan=All years |
|  | #3 | TOPIC: ("Outcome Assessment, Health Care") OR TOPIC: ("Outcomes Assessment") OR TOPIC: ("Outcome Assessment (Health Care) ") OR TOPIC: ("Assessment, Outcome (Health Care) ") OR TOPIC: ("Assessments, Outcome (Health Care) ") OR TOPIC: ("Outcome Assessments (Health Care) ") OR TOPIC: ("Assessment, Outcomes") OR TOPIC: ("Assessments, Outcomes") OR TOPIC: ("Outcomes Assessments") OR TOPIC: ("Outcomes Research") OR TOPIC: ("Research, Outcomes") OR TOPIC: ("Outcome Studies") OR TOPIC: ("Outcome Study") OR TOPIC: ("Studies, Outcome") OR TOPIC: ("Study, Outcome") OR TOPIC: ("Outcome Measures") OR TOPIC: ("Measure, Outcome") OR TOPIC: ("Measures, Outcome") OR TOPIC: ("Outcome Measure") OR TOPIC: ("Outcome") OR TOPIC: ("Outcomes") OR TOPIC: ("Result") OR TOPIC: ("Results") OR TOPIC: ("Health outcome") OR TOPIC: ("Health outcomes")  Indexes=SCI-EXPANDED, SSCI, A&HCI, CPCI-S, CPCI-SSH, ESCI Timespan=All years |
|  | #4 | TOPIC: ("Therapeutic outcome") OR TOPIC: ("Outcome Assessment, Health Care")  Indexes=SCI-EXPANDED, SSCI, A&HCI, CPCI-S, CPCI-SSH, ESCI Timespan=All years |
|  | #5 | #4 OR #3  Indexes=SCI-EXPANDED, SSCI, A&HCI, CPCI-S, CPCI-SSH, ESCI Timespan=All years |
|  | #6 | TOPIC: ("Pharmacists") OR TOPIC: ("Pharmacist") OR TOPIC: ("Clinical Pharmacists") OR TOPIC: ("Clinical Pharmacist") OR TOPIC: ("Pharmacist, Clinical") OR TOPIC: ("Pharmacists, Clinical") OR TOPIC: ("Community Pharmacists") OR TOPIC: ("Community Pharmacist") OR TOPIC: ("Pharmacist, Community") OR TOPIC: ("Pharmacists, Community") OR TOPIC: ("Retail Pharmacists") OR TOPIC: ("Pharmacist, Retail") OR TOPIC: ("Pharmacists, Retail") OR TOPIC: ("Retail Pharmacist") OR TOPIC: ("community pharmacy services") OR TOPIC: ("Pharmaceutical Service, Community") OR TOPIC: ("Pharmaceutical Services, Community") OR TOPIC: ("Service, Community Pharmaceutical") OR TOPIC: ("Services, Community Pharmaceutical") OR TOPIC: ("Pharmacy Services, Community") OR TOPIC: ("Community Pharmacy Service") OR TOPIC: ("Pharmacy Service, Community") OR TOPIC: ("Services, Community Pharmaceutic") OR TOPIC: ("Services, Community Pharmacy") OR TOPIC: ("Community Pharmaceutic Services")  Indexes=SCI-EXPANDED, SSCI, A&HCI, CPCI-S, CPCI-SSH, ESCI Timespan=All years |
|  | #7 | TOPIC: ("Community Pharmaceutic Service") OR TOPIC: ("Pharmaceutic Service, Community") OR TOPIC: ("Pharmaceutic Services, Community") OR TOPIC: ("Service, Community Pharmaceutic") OR TOPIC: ("Community Pharmaceutical Services") OR TOPIC: ("Community Pharmaceutical Service") OR TOPIC: ("Service, Community Pharmacy") OR TOPIC: ("pharmacies") OR TOPIC: ("Pharmacy Distribution") OR TOPIC: ("Distribution, Pharmacy") OR TOPIC: ("Distributions, Pharmacy") OR TOPIC: ("Pharmacy Distributions") OR TOPIC: ("Community Pharmacies") OR TOPIC: ("Community Pharmacy") OR TOPIC: ("Pharmacies, Community") OR TOPIC: ("Pharmacy, Community")  Indexes=SCI-EXPANDED, SSCI, A&HCI, CPCI-S, CPCI-SSH, ESCI Timespan=All years |
|  | #8 | #7 OR #6  Indexes=SCI-EXPANDED, SSCI, A&HCI, CPCI-S, CPCI-SSH, ESCI Timespan=All years |
|  | #9 | #8 AND #5 AND #2 AND #1  Indexes=SCI-EXPANDED, SSCI, A&HCI, CPCI-S, CPCI-SSH, ESCI Timespan=All year |

*From*: Page MJ, McKenzie JE, Bossuyt PM, Boutron I, Hoffmann TC, Mulrow CD, et al. The PRISMA 2020 statement: an updated guideline for reporting systematic reviews. BMJ 2021;372:n71. doi: 10.1136/bmj.n71. For more information, visit: http://www.prisma-statement.org/
