# Supplementary material for: Does drug dispensing improve the health outcomes of patients attending community pharmacies? A systematic review
Source: BMC Health Serv Res. 2021 Aug 2;21:764. doi: 10.1186/s12913-021-06770-0 (PMC8330087; doi:10.1186/s12913-021-06770-0)
Supplement: Supplementary file 2 — Additional file 2. List of excluded articles and reason for exclusion. [file 12913_2021_6770_MOESM2_ESM.docx]

**Does drug dispensing improve the health outcomes of patients attending community pharmacies? A systematic review**

**Bárbara Pizetta**

Research Group on Implementation and Integration of Clinical Pharmacy Services in Brazilian Health System (SUS), Department of Pharmacy and Nutrition, Federal University of Espírito Santo, Alegre, ES, Brazil.

E-mail: pizetta.barbara@gmail.com

**Lívia Gonçalves Raggi**

Research Group on Implementation and Integration of Clinical Pharmacy Services in Brazilian Health System (SUS), Department of Pharmacy and Nutrition, Federal University of Espírito Santo, Alegre, ES, Brazil.

E-mail: livia_gr@hotmail.com

**Kérilin Stancine Santos Rocha**

Health Sciences Graduate Program

Laboratory of Teaching and Research in Social Pharmacy (LEPFS), Department of Pharmacy, Federal University of Sergipe, São Cristóvão, SE, Brazil.

E-mail: kerilin.farm@gmail.com

**Sabrina Cerqueira Santos**

Graduate Program in Pharmaceutical Sciences

Laboratory of Teaching and Research in Social Pharmacy (LEPFS), Department of Pharmacy, Federal University of Sergipe, São Cristóvão, SE, Brazil.

E-mail: Sabrina-cerqueira@hotmail.com

**Divaldo Pereira de Lyra Jr**

Laboratory of Teaching and Research in Social Pharmacy (LEPFS), Department of Pharmacy, Federal University of Sergipe, São Cristóvão, SE, Brazil.

E-mail: lepfs.ufs@gmail.com

**Genival Araujo dos Santos Júnior***

Research Group on Implementation and Integration of Clinical Pharmacy Services in Brazilian Health System (SUS), Department of Pharmacy and Nutrition, Federal University of Espírito Santo, Alegre, ES, Brazil.

E-mail: farm.genival@gmail.com

***Corresponding author:**

**Genival Araujo dos Santos Júnior***

Research Group on Implementation and Integration of Clinical Pharmacy Services in Brazilian Health System (SUS), Department of Pharmacy and Nutrition, Federal University of Espírito Santo, Alegre, ES, Brazil.

E-mail: [farm.genival@gmail.com](about:blank)

**APPENDIX B - List of excluded articles and reason for exclusion**

| **Title** | **Author, year** | **Reason for exclusion** |
| --- | --- | --- |
| A community-pharmacy-based callback program for antibiotic therapy | Westfall et al., 1997 | It's not a dispensing |
| A comparison of the Indian Health Service counseling technique with traditional, lecture-style counseling | Lam et al., 2015 | Does not address patient health outcomes |
| A multifactorial intervention to enhance adherence to medications and disease-related knowledge in type 2 diabetic patients in Southern Punjab, Pakistan | Samtia et al., 2013 | It's not a community pharmacy |
| A quantitative evaluation of adherence and inhalation technique among respiratory patients: An observational study using an electronic inhaler assessment device | Hesso et al., 2020 | It's not a dispensing |
| A randomised controlled trial of the impact of structured written and verbal advice by community pharmacists on improving hypertension education and control in patients with high blood pressure | Cheema et al., 2018 | It's not a dispensing |
| A randomized controlled study on the effectiveness of community pharmacists' advice for the smoking cessation by Nicorette - Evaluation at three months after starting the cessation | Mochizuki et al., 2004 | Language |
| A randomized study to assess the impact of pharmacist counseling of employer-based health plan beneficiaries with diabetes: the EMPOWER study | Kraemer. et al., 2012 | It's not a dispensing |
| Algorithm of proceedings in the community pharmacy to optimize the use of statins | Ferrer Estrela et al., 2015 | Results do not separate dispensing from other services /interventions |
| Assessment of patient's satisfaction with pharmaceutical care services in community pharmacies in the United Arab Emirates | El-Sharif et al., 2017 | Results do not separate dispensing from other services /interventions |
| **Title** | **Author, year** | **Reason for exclusion** |
| Asthma patients' satisfaction with the frequency and content of pharmacist counseling | Liu et al., 1999 | It's not a community pharmacy |
| Australian mental health consumers' and carers' experiences of community pharmacy service | Knox et al., 2015 | Results do not separate dispensing from other services /interventions |
| Clinical pharmacy interventions by community pharmacists during the dispensing process | Hawksworth et al., 1999 | Does not address patient health outcomes |
| Clinical risk management in dutch community pharmacies the case of drug-drug interactions | Buurma et al., 2006 | Does not address patient health outcomes |
| Continuous Medication Monitoring (CoMM): A foundational model to support the clinical work of community pharmacists | Goedken et al., 2018 | Results do not separate the intervention of the pharmacist from the intervention of other professionals |
| Diabetes Medication Assistance Service: the pharmacist's role in supporting patient self-management of type 2 diabetes (T2DM) in Australia | Mitchell et al., 2011 | It's not a dispensing |
| Drug related problems identified by European community pharmacists in patients discharged from hospital | Paulino et al., 2004 | Results do not separate dispensing from other services /interventions |
| Drug related problems in Belgian community pharmacies | Huysmans et al., 2014 | Language |
| Drug-related problems in Parkinson's disease: the role of community pharmacists in primary care | Schröder et al., 2011 | Results do not separate dispensing from other services /interventions |
| Drug-related problems in prescribed medicines in Germany at the time of dispensing | Nicolas et al., 2013 | Does not address patient health outcomes |
| Effectiveness of interventions by community pharmacists to reduce risk of gastrointestinal side effects in nonselective nonsteroidal anti-inflammatory drug users | Teichert et al., 2014 | Results do not separate dispensing from other services /interventions |
| Effect of a pharmacy care program on medication adherence and persistence, blood pressure, and low-density lipoprotein cholesterol: a randomized controlled trial | Lee et al., 2006 | It's not a community pharmacy |
| Effectiveness of a protocolized dispensing service in community pharmacy for improving patient medication knowledge | Abaurre-Labrador et al., 2016 | Does not address patient health outcomes |
| **Title** | **Author, year** | **Reason for exclusion** |
| Effect of patient counseling on quality of life in type-2 diabetes mellitus patients in two selected South Indian community pharmacies: a study | Adepu et al., 2007 | Results do not separate dispensing from other services /interventions |
| Effects of lifestyle advice provided by pharmacists on blood pressure: The COMmunity Pharmacists ASSist for Blood Pressure (COMPASS-BP) randomized trial | Okada et al., 2017 | It's not a dispensing |
| Emergency contraception counseling in a retail pharmacy setting: a pilot study | Ragland et al., 2015 | It's not a dispensing |
| Evaluation of a model for counseling patients with dyspepsia in Swedish community pharmacies | Westerlund et al., 2003 | Results do not separate the intervention of the pharmacist from the intervention of other professionals |
| Evaluation of the impact of pharmacist's advice giving on the outcomes of self-medication in patients suffering from dyspepsia | Krishnan et al., 2000 | It's not a dispensing |
| Exploring patient expectations for pharmacist-provided literacy-sensitive communication | Collum et al., 2013 | It's not a dispensing |
| Exploring the extended role of the community pharmacist in improving blood pressure control among hypertensive patients in a developing setting | Marfo et al., 2017 | Results do not separate dispensing from other services /interventions |
| Extended adherence support by community pharmacists for patients with hypertension: a randomised controlled trial | Blenkinsopp et al., 2000 | Results do not separate dispensing from other services /interventions |
| Family-based intervention by pharmacists for type 2 diabetes: a randomised controlled trial | Withidpanyawong et al., 2019 | It's not a community pharmacy |
| Health-Related Quality of Life after Ischemic Stroke: The Impact of Pharmaceutical Interventions on Drug Therapy (Pharmaceutical Care Concept) | Hohmann et al., 2010 | Results do not separate dispensing from other services /interventions |
| Impact of a pharmaceutical care intervention on blood pressure control in a chain pharmacy practice | Robinson et al., 2010 | Results do not separate dispensing from other services /interventions |
| **Title** | **Author, year** | **Reason for exclusion** |
| Impact of community pharmacists' interventions on asthma self-management care | Kovačević et al., 2018 | It's not a dispensing |
| Impact of the pay-for-performance-for-patients program for diabetes management | Hui-Callahan et al., 2013 | Results do not separate dispensing from other services /interventions |
| Impact of the pharmaceutical intervention performed on cancer patients in community pharmacy | De Lecea et al., 2020 | Results do not separate dispensing from other services /interventions |
| Medication adherence and clinical outcomes in dispensing and non-dispensing practices: a cross-sectional analysis | Gomez-cano et al., 2021 | It's not a community pharmacy |
| Medication dispensing as an opportunity for patient counseling and approach to drug-related problems | Ferreira et al., 2016 | Does not address patient health outcomes |
| Outcomes of a community pharmacy-based diabetes monitoring program | Berringer et al., 1999 | Results do not separate dispensing from other services /interventions |
| Patient counselling service with the use of pictograms as the example of pharmacist intervention to improving compliance and medicine safety | Merks et al., 2021 | Does not address patient health outcomes |
| Patients' perceived benefit from and satisfaction with asthma-related pharmacy services | Kradjan et al., 1999 | Results do not separate dispensing from other services /interventions |
| Patients' perception, views and satisfaction with pharmacists' role as health care provider in community pharmacy setting at Riyadh, Saudi Arabia | Al-Arifi, 2012 | Does not address patient health outcomes |
| Pharmaceutical care for migraine and headache patients: a community-based, randomized intervention | Hoffmann et al., 2008 | It's not a dispensing |
| Pharmaceutical care for patients with ischemic stroke: improving the patients quality of life | Hohmann et al., 2009 | Results do not separate dispensing from other services /interventions |
| Pharmaceutical interventions on prescribed medicines in community pharmacies : focus on patient ‑ reported problems | Maes et al., 2018 | Does not address patient health outcomes |
| Pharmacist competences and impact of pharmacist intervention on medication adherence : an observational study | Boskovic et al., 2016 | Does not address patient health outcomes |
| **Title** | **Author, year** | **Reason for exclusion** |
| Pharmacist counseling in a cohort of women with HIV and women at risk for HIV | Cocohoba et al., 2012 | Results do not separate dispensing from other services /interventions |
| Pharmacist intervention reduces gastropathy risk in patients using NSAIDs | Ibañez-Cuevas et al., 2008 | Results do not separate dispensing from other services /interventions |
| Pharmacist-led intervention study to improve drug therapy in asthma and COPD patients | Ottenbros et al., 2014 | Results do not separate dispensing from other services /interventions |
| Pharmacy performance while providing continuous medication monitoring | Doucette et al., 2017 | Results do not separate dispensing from other services/interventions |
| Preventable drug-related morbidity in community pharmacy: development and piloting of a complex intervention | Guerreiro et al., 2012 | Results do not separate dispensing from other services/interventions |
| Safe and effective use of medicines for patients with type 2 diabetes - A randomized controlled trial of two interventions delivered by local pharmacies | Kjeldsen et al., 2015 | Results do not separate dispensing from other services/interventions |
| Survey of drug-related problems identified by community pharmacies | Haemmerlein et al., 2009 | Results do not separate dispensing from other services/interventions |
| The effectiveness of counseling using preventative informational sheets with climate and environmental data from insurance pharmacies in preventing worsened asthma symptoms | Iwade et al., 2019 | Language |
| The impact of pharmacist face-to-face counseling to improve medication adherence among patients initiating statin therapy | Taitel et al., 2012 | Does not address patient health outcomes |
| Uptake and effectiveness of a community pharmacy intervention programme to improve asthma management | Bereznicki et al., 2013 | It's not a dispensing |
| Value of community pharmacists' interventions to correct prescribing errors | Rupp, 1992 | Does not address patient health outcomes |

*From:*  Page MJ, McKenzie JE, Bossuyt PM, Boutron I, Hoffmann TC, Mulrow CD, et al. The PRISMA 2020 statement: an updated guideline for reporting systematic reviews. BMJ 2021;372:n71. doi: 10.1136/bmj.n71. For more information, visit: <http://www.prisma-statement.org/>
