# Supplementary material for: Does drug dispensing improve the health outcomes of patients attending community pharmacies? A systematic review
Source: BMC Health Serv Res. 2021 Aug 2;21:764. doi: 10.1186/s12913-021-06770-0 (PMC8330087; doi:10.1186/s12913-021-06770-0)
Supplement: Supplementary file 3 — Additional file 3. Assessment of methodological quality [file 12913_2021_6770_MOESM3_ESM.docx]

**Does drug dispensing improve the health outcomes of patients attending community pharmacies? A systematic review**

**Bárbara Pizetta**

Research Group on Implementation and Integration of Clinical Pharmacy Services in Brazilian Health System (SUS), Department of Pharmacy and Nutrition, Federal University of Espírito Santo, Alegre, ES, Brazil.

E-mail: pizetta.barbara@gmail.com

**Lívia Gonçalves Raggi**

Research Group on Implementation and Integration of Clinical Pharmacy Services in Brazilian Health System (SUS), Department of Pharmacy and Nutrition, Federal University of Espírito Santo, Alegre, ES, Brazil.

E-mail: livia_gr@hotmail.com

**Kérilin Stancine Santos Rocha**

Health Sciences Graduate Program

Laboratory of Teaching and Research in Social Pharmacy (LEPFS), Department of Pharmacy, Federal University of Sergipe, São Cristóvão, SE, Brazil.

E-mail: kerilin.farm@gmail.com

**Sabrina Cerqueira Santos**

Graduate Program in Pharmaceutical Sciences

Laboratory of Teaching and Research in Social Pharmacy (LEPFS), Department of Pharmacy, Federal University of Sergipe, São Cristóvão, SE, Brazil.

E-mail: Sabrina-cerqueira@hotmail.com

**Divaldo Pereira de Lyra Jr**

Laboratory of Teaching and Research in Social Pharmacy (LEPFS), Department of Pharmacy, Federal University of Sergipe, São Cristóvão, SE, Brazil.

E-mail: lepfs.ufs@gmail.com

**Genival Araujo dos Santos Júnior***

Research Group on Implementation and Integration of Clinical Pharmacy Services in Brazilian Health System (SUS), Department of Pharmacy and Nutrition, Federal University of Espírito Santo, Alegre, ES, Brazil.

E-mail: farm.genival@gmail.com

***Corresponding author:**

**Genival Araujo dos Santos Júnior***

Research Group on Implementation and Integration of Clinical Pharmacy Services in Brazilian Health System (SUS), Department of Pharmacy and Nutrition, Federal University of Espírito Santo, Alegre, ES, Brazil.

E-mail: [farm.genival@gmail.com](about:blank)

**Appendix C - Assessment of methodological quality**

Table 1. Quality assessment for cross-sectional studies using the tool “JBI Critical Appraisal Checklist for Analytical Cross Sectional Studies”

| **Criteria** | **Chong et al., 2011** | **Ali et al., 2019** | **Payne et al.,**  **2019** | **Westerlund et al., 2009** |
| --- | --- | --- | --- | --- |
| 1. Were the criteria for inclusion in the sample clearly defined? | No | Yes | No | Unclear |
| 2. Were the study subjects and the setting described in detail? | Yes | Yes | No | Yes |
| 3. Was the exposure measured in a valid and reliable way? | Unclear | Unclear | Unclear | Unclear |
| 4. Were objective, standard criteria used for measurement of the condition? | Yes | Yes | Unclear | Yes |
| 5. Were confounding factors identified? | Yes | Yes | No | No |
| 6. Were strategies to deal with confounding factors stated? | Yes | No | No | No |
| 7. Were the outcomes measured in a valid and reliable way? | Unclear | Yes | Unclear | Unclear |
| 8. Was appropriate statistical analysis used? | Yes | Yes | No | Unclear |

Table 2. Quality assessment for quasi-experimental studies using the tool “JBI Critical Appraisal Checklist for Quasi-Experimental Studies”.

| **Criteria** | **Ferreira et al., 2018** |
| --- | --- |
| 1. Is it clear in the study what is the ‘cause’ and what is the ‘effect’ (i.e. there is no confusion about which variable comes first)? | Yes |
| 2. Were the participants included in any comparisons similar? | Yes |
| 3. Were the participants included in any comparisons receiving similar treatment/care, other than the exposure or intervention of interest? | Unclear |
| 4. Was there a control group? | No |
| 5. Were there multiple measurements of the outcome both pre and post the intervention/exposure? | Yes |
| 6. Was follow up complete and if not, were differences between groups in terms of their follow up adequately described and analyzed? | Unclear |
| 7. Were the outcomes of participants included in any comparisons measured in the same way? | Yes |
| 8. Were outcomes measured in a reliable way? | Unclear |
| 9. Was appropriate statistical analysis used? | Yes |

Table 3. Quality assessment for randomized clinical trials using the “JBI Critical Appraisal Checklist for Randomized Controlled Trials” tool.

| **Criteria** | **Crockett et al., 2006** | **Merks et al., 2019** | **Basheti et al., 2008** | **O’Dwyer et al., 2020** |
| --- | --- | --- | --- | --- |
| 1. Was true randomization used for assignment of participants to treatment groups? | Unclear | Unclear | Yes | Yes |
| 2. Was allocation to treatment groups concealed? | Unclear | Unclear | Unclear | Unclear |
| 3. Were treatment groups similar at the baseline? | Yes | Yes | Unclear | Unclear |
| 4. Were participants blind to treatment assignment? | Unclear | Unclear | Unclear | No |
| 5. Were those delivering treatment blind to treatment assignment? | No | Unclear | Yes | No |
| 6. Were outcomes assessors blind to treatment assignment? | Unclear | Unclear | No | Unclear |
| 7. Were treatment groups treated identically other than the intervention of interest? | Unclear | Unclear | Unclear | Unclear |
| 8. Was follow up complete and if not, were differences between groups in terms of their follow up adequately described and analyzed? | No | No | No | No |
| 9. Were participants analyzed in the groups to which they were randomized? | No | No | Unclear | Yes |
| 10. Were outcomes measured in the same way for treatment groups? | Yes | Yes | Yes | Yes |
| 11. Were outcomes measured in a reliable way? | Unclear | Unclear | Unclear | Yes |

Table 4. Quality assessment for randomized clinical trials using the “JBI Critical Appraisal Checklist for Randomized Controlled Trials” tool (continuation).

| **Criteria** | **Crockett et al., 2006** | **Merks et al., 2019** | **Basheti et al., 2008** | **O’Dwyer et al., 2020** |
| --- | --- | --- | --- | --- |
| 12. Was appropriate statistical analysis used? | Yes | Yes | Yes | Yes |
| 13. Was the trial design appropriate, and any deviations from the standard RCT design (individual randomization, parallel groups) accounted for in the conduct and analysis of the trial? | No | Unclear | Unclear | Yes |
